# (2*E*)-3-(3-Bromo-4-meth­oxy­phen­yl)-1-(4-methyl­phen­yl)prop-2-en-1-one

**DOI:** 10.1107/S1600536811011482

**Published:** 2011-03-31

**Authors:** Grzegorz Dutkiewicz, B. P. Siddaraju, H. S. Yathirajan, B. Narayana, Maciej Kubicki

**Affiliations:** a Department of Chemistry, Adam Mickiewicz University, Grunwaldzka 6 60-780, Poznań, Poland; b Department of Studies in Chemistry, University of Mysore, Manasagangotri, Mysore 570 006, India; c Department of Studies in Chemistry, Mangalore University, Mangalagangotri, 574 199 India

## Abstract

The overall shape of the mol­ecule of the title compound, C_17_H_15_BrO_2_, can be described by the dihedral angles between three planar fragments: 1-bromo-2-meth­oxy­phenyl ring [maximum deviation = 0.003 (2) Å], the central prop-2-en-1-one chain [maximum deviation = 0.005 (2) Å], and the methyl­phenyl ring [maximum deviation = 0.004 (2) Å]. The terminal planes are twisted by 10.37 (12)°, while the central plane is almost coplanar with the methyl­phenyl ring [3.30 (13)°], but the dihedral angle with the other phenyl ring is significantly larger [8.76 (16)°]. In the crystal, mol­ecules are linked into chains along [001] by three C—H⋯O hydrogen bonds. These chains inter­act with each other by means of weak π–π contacts [centroid–centroid distances = 3.73 (1) and 3.44 (1) Å]. An inter­molecular C—H⋯Br inter­action also occurs.

## Related literature

For related structures, see: Butcher *et al.* (2006 ▶); Ng *et al.* (2006 ▶); Zhou (2010 ▶). For a description of the Cambridge Structural Database, see: Allen (2002 ▶).
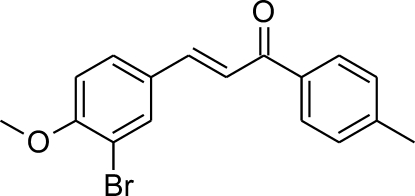


## Experimental

### 

#### Crystal data

C_17_H_15_BrO_2_
*M* *_r_* = 331.19
Monoclinic, 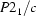

*a* = 11.680 (2) Å
*b* = 11.654 (2) Å
*c* = 10.834 (2) Å
β = 93.07 (2)°
*V* = 1472.6 (4) Å^3^
*Z* = 4
Mo *K*α radiation
μ = 2.79 mm^−1^
*T* = 295 K
0.5 × 0.3 × 0.1 mm

#### Data collection

Agilent Xcalibur Eos diffractometer
Absorption correction: multi-scan (*CrysAlis PRO*; Agilent, 2010 ▶) *T*_min_ = 0.276, *T*_max_ = 1.000
6000 measured reflections
3003 independent reflections
1455 reflections with *I* > 2σ(*I*)
*R*_int_ = 0.030

#### Refinement

*R*[*F* ^2^ > 2σ(*F* ^2^)] = 0.043
*wR*(*F* ^2^) = 0.072
*S* = 1.00
3003 reflections
183 parameters
H-atom parameters constrained
Δρ_max_ = 0.44 e Å^−3^
Δρ_min_ = −0.44 e Å^−3^

### 

Data collection: *CrysAlis PRO* (Agilent, 2010 ▶); cell refinement: *CrysAlis PRO*; data reduction: *CrysAlis PRO*; program(s) used to solve structure: *SIR92* (Altomare *et al.*, 1993 ▶); program(s) used to refine structure: *SHELXL97* (Sheldrick, 2008 ▶); molecular graphics: *Stereochemical Workstation Operation Manual* (Siemens, 1989 ▶); software used to prepare material for publication: *SHELXL97*.

## Supplementary Material

Crystal structure: contains datablocks I, global. DOI: 10.1107/S1600536811011482/rk2271sup1.cif

Structure factors: contains datablocks I. DOI: 10.1107/S1600536811011482/rk2271Isup2.hkl

Additional supplementary materials:  crystallographic information; 3D view; checkCIF report

## Figures and Tables

**Table 1:** Hydrogen-bond geometry (Å, °)

*D*—H⋯*A*	*D*—H	H⋯*A*	*D*⋯*A*	*D*—H⋯*A*
C2—H2⋯O1^i^	0.93	2.61	3.536 (3)	178
C5—H5⋯O1^i^	0.93	2.69	3.603 (4)	167
C12—H12⋯O1^i^	0.93	2.50	3.424 (4)	171
C141—H14*B*⋯Br6^ii^	0.96	3.14	4.100 (3)	176

Symmetry codes: (i) 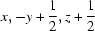; (ii) 
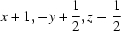
.

**Fig. 1.**
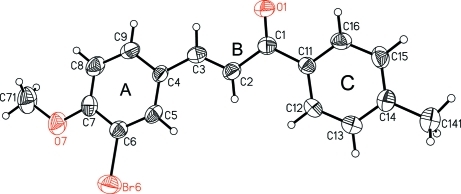
Anisotropic ellipsoid representation of the compound I together with atom labeling scheme. Displacement ellipsoids are drawn at 50% probability level. Hydrogen atoms are shown as spheres of arbitrary radius.

**Fig. 2.**
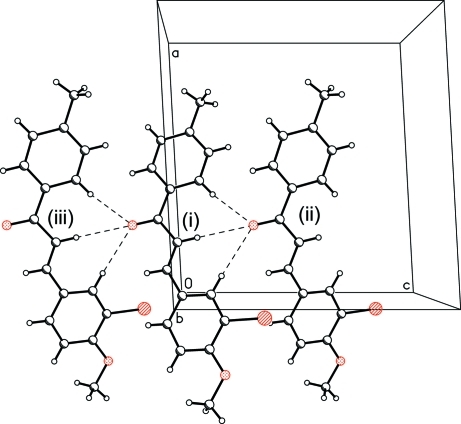
The hydrogen - bonded chain of molecules of I. Hydrogen bonds are shown as dashed lines. Symmetry codes: (i) x, y, z; (ii) x, 1/2-y, 1/2+z; (iii) x, 1/2-y, -1/2+z.

**Fig. 3.**
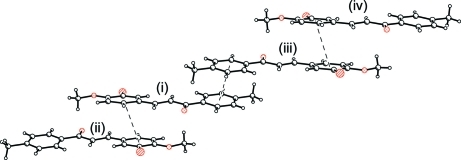
The π···π interactions (shown as dashed lines between the centroids of the phenyl rings) between the neighbouring chains. Symmetry codes: (i) x, y, z; (ii) -x,1-y, -z; (iii) 1-x, -y, -z; (iv) 1+x, -1+y, z.

## supplementary crystallographic information

### Comment

Chalcone (1,3-diphenyl-2-propen-1-one) derivatives and their heterocyclic analogues are valuable intermediates in organic synthesis and exhibit a wide range of biological activities, as well as non-linear optical properties with excellent blue light transmittance and good crystallizability. As a part of our ongoing studies in the chalcone structural chemistry we have synthesized a new chalcone (2*E*)-3-(3-bromo-4-methoxyphenyl)-1-(4-methylphenyl)prop-2-en-1-one (I, Scheme 1). The packing of the molecules in crystals is a result of the compromise between different intermolecular interactions, tendency towards close packing, symmetry requirements *etc*. Therefore, studying of the crystal packing might be useful in the understanding of different intermolecular interactions. In the absence of potential good hydrogen bond donors - as it is the case of the molecule described here - the crystal structure might be determined by other interactions and requirements: close packing, van der Waals interactions, weak hydrogen bonds (C—H···O, Br, or π), halogen bonds - as there are both C—Br and C═O groups available, π···π stacking *etc*.

The overall shape of the molecule can be described by the dihedral angles between three planar fragments (*cf.* Fig. 1): 1-bromo-2-methoxyphenyl ring (A), the central prop-2-en-1-one chain (B), and 4-methylphenyl ring (C). The terminal planes A and C are twisted by 10.37 (12)°, while the central plane B is almost coplanar with C (3.30 (13)°), and the dihedral angle with A is significantly larger, 8.76 (16)°. The similar molecules found in the Cambridge Crystallographic Database (Allen, 2002) differ significantly in their conformations, thus suggesting that it depends mostly on the intermolecular interactions. For instance - limiting to similar pattern of substitution - in 3-(3,4-dimethoxyphenyl)-1-(4-fluorophenyl)-prop-2-en-1-one (Butcher *et al.*, 2006) A/C dihedral angle is 47.81 (6)° and 50.18 (5)° in two symmety independent molecules, while in 3-(3,4-dimethoxyphenyl)-1-(4-bromophenyl)-prop-2-en-1-one (Ng *et al.*, 2006) there are also two symmetry-independent molecules, but there are almost planar, the dihedral angles between the phenyl rings are 9.30 (15)° and 4.85 (16)°. Again in 3-(3,4-dimethylphenyl)-1-(4-bromophenyl)-prop-2-en-1-one (Zhou, 2010) the twist is significant, 48.13 (4)°.

In the structure of I quite a rich structure of weak interactions can be found. The molecules are connected into chains along [0 0 1] direction by means of three C—H···O hydrogen bonds (Table 1, Fig. 2). These chains are interacting with the other ones by means of weak π···π contacts. The centroid-to-centroid distances are *Cg*A···*Cg*A 3.729Å and *Cg*B···*Cg*B 3.748Å, which - taking into account the slippage - translates into the interplanar distances of *ca*. 3.52Å for A···A contacts and 3.44Å for B···B ones (Fig. 3).

### Experimental

3-Bromo-4-methoxybenzaldehyde (2.15 g, 0.01 mol) was mixed with 1-(4-methylphenyl) ethanone (1.34 g, 0.01 mol) and dissolved in ethanol (40 ml). To the solution, 4 ml of KOH (50%) was added. The reaction mixture was stirred for 6–10 h. The resulting crude solid was filtered, washed successively with distilled water and finally recrystallized from ethanol (95%) to give the pure chalcones. Crystals suitable for X-ray diffraction studies were grown by the slow evaporation from acetone solution (m.p. = 393 K). Composition: Found (Calculated): C_17_H_15_BrO_2_ - C: 61.58 (61.65); H: 4.51 (4.56).

### Refinement

The hydrogen were placed geometrically, in idealized positions, and refined as rigid groups with their *U*_iso_(H) = 1.2*U*_eq_(C) with distances C—H = 0.93Å of the appropriate carrier atom (*U*_iso_(H) = 1.5*U*_eq_(C) with distances C—H = 0.96Å for methyl H).

### Crystal data

**Table:**

C_17_H_15_BrO_2_	*F*(000) = 672
*M**_r_* = 331.19	*D*_x_ = 1.494 Mg m^−^^3^
Monoclinic, *P*2_1_/*c*	Melting point: 393 K
Hall symbol: -P 2ybc	Mo *K*α radiation, λ = 0.71073 Å
*a* = 11.680 (2) Å	Cell parameters from 2134 reflections
*b* = 11.654 (2) Å	θ = 3.1–27.9°
*c* = 10.834 (2) Å	µ = 2.79 mm^−^^1^
β = 93.07 (2)°	*T* = 295 K
*V* = 1472.6 (4) Å^3^	Plate, colourless
*Z* = 4	0.5 × 0.3 × 0.1 mm

### Data collection

**Table:**

Agilent Xcalibur Eos diffractometer	3003 independent reflections
Radiation source: Enhance (Mo) X-ray Source	1455 reflections with *I* > 2σ(*I*)
graphite	*R*_int_ = 0.030
Detector resolution: 16.1544 pixels mm^-1^	θ_max_ = 28.0°, θ_min_ = 3.1°
ω–scan	*h* = −9→15
Absorption correction: multi-scan (*CrysAlis PRO*; Agilent, 2010)	*k* = −13→9
*T*_min_ = 0.276, *T*_max_ = 1.000	*l* = −12→14
6000 measured reflections	

### Refinement

**Table:**

Refinement on *F*^2^	Primary atom site location: structure-invariant direct methods
Least-squares matrix: full	Secondary atom site location: difference Fourier map
*R*[*F*^2^ > 2σ(*F*^2^)] = 0.043	Hydrogen site location: inferred from neighbouring sites
*wR*(*F*^2^) = 0.072	H-atom parameters constrained
*S* = 1.00	*w* = 1/[σ^2^(*F*_o_^2^) + (0.025*P*)^2^] where *P* = (*F*_o_^2^ + 2*F*_c_^2^)/3
3003 reflections	(Δ/σ)_max_ = 0.001
183 parameters	Δρ_max_ = 0.44 e Å^−^^3^
0 restraints	Δρ_min_ = −0.44 e Å^−^^3^

### Special details

**Table:**

Geometry. All s.u.'s (except the s.u. in the dihedral angle between two l.s. planes) are estimated using the full covariance matrix. The cell s.u.'s are taken into account individually in the estimation of s.u.'s in distances, angles and torsion angles; correlations between s.u.'s in cell parameters are only used when they are defined by crystal symmetry. An approximate (isotropic) treatment of cell s.u.'s is used for estimating s.u.'s involving l.s. planes.
Refinement. Refinement of *F*^2^ against ALL reflections. The weighted *R*-factor *wR* and goodness of fit *S* are based on *F*^2^, conventional *R*-factors *R* are based on *F*, with *F* set to zero for negative *F*^2^. The threshold expression of *F*^2^ > σ(*F*^2^) is used only for calculating *R*-factors(gt) *etc*. and is not relevant to the choice of reflections for refinement. *R*-factors based on *F*^2^ are statistically about twice as large as those based on *F*, and *R*-factors based on ALL data will be even larger.

### Fractional atomic coordinates and isotropic or equivalent isotropic displacement parameters (Å^2^)

**Table:**

	*x*	*y*	*z*	*U*_iso_*/*U*_eq_	
C1	0.2928 (3)	0.2026 (2)	−0.0764 (3)	0.0370 (8)	
O1	0.27055 (17)	0.18029 (18)	−0.18486 (19)	0.0502 (6)	
C2	0.2054 (2)	0.2537 (2)	0.0006 (3)	0.0389 (8)	
H2	0.2235	0.2686	0.0837	0.047*	
C3	0.1015 (3)	0.2784 (2)	−0.0473 (3)	0.0387 (8)	
H3	0.0881	0.2593	−0.1302	0.046*	
C4	0.0056 (3)	0.3310 (2)	0.0112 (3)	0.0336 (7)	
C5	0.0129 (3)	0.3788 (2)	0.1281 (3)	0.0376 (8)	
H5	0.0823	0.3762	0.1744	0.045*	
C6	−0.0804 (3)	0.4300 (3)	0.1771 (3)	0.0379 (8)	
Br6	−0.06520 (3)	0.50120 (3)	0.33361 (3)	0.06471 (15)	
C7	−0.1850 (3)	0.4354 (3)	0.1113 (3)	0.0394 (8)	
O7	−0.27311 (18)	0.48560 (18)	0.1679 (2)	0.0561 (6)	
C71	−0.3790 (3)	0.4986 (3)	0.0993 (3)	0.0688 (10)	
H71A	−0.3664	0.5362	0.0224	0.103*	
H71B	−0.4301	0.5440	0.1459	0.103*	
H71C	−0.4124	0.4245	0.0833	0.103*	
C8	−0.1948 (3)	0.3877 (3)	−0.0058 (3)	0.0477 (9)	
H8	−0.2645	0.3897	−0.0515	0.057*	
C9	−0.0998 (3)	0.3367 (3)	−0.0546 (3)	0.0462 (9)	
H9	−0.1068	0.3054	−0.1336	0.055*	
C11	0.4101 (3)	0.1804 (2)	−0.0206 (3)	0.0325 (7)	
C12	0.4420 (3)	0.1998 (2)	0.1032 (3)	0.0466 (9)	
H12	0.3886	0.2285	0.1560	0.056*	
C13	0.5524 (3)	0.1766 (3)	0.1483 (3)	0.0490 (9)	
H13	0.5720	0.1893	0.2315	0.059*	
C14	0.6338 (3)	0.1350 (2)	0.0731 (3)	0.0404 (9)	
C141	0.7531 (3)	0.1098 (3)	0.1243 (3)	0.0591 (10)	
H14A	0.7866	0.1784	0.1594	0.089*	
H14B	0.7988	0.0828	0.0591	0.089*	
H14C	0.7503	0.0519	0.1871	0.089*	
C15	0.6026 (3)	0.1157 (2)	−0.0504 (3)	0.0444 (9)	
H15	0.6562	0.0877	−0.1032	0.053*	
C16	0.4931 (3)	0.1376 (2)	−0.0946 (3)	0.0420 (9)	
H16	0.4736	0.1233	−0.1775	0.050*	

### Atomic displacement parameters (Å^2^)

**Table:**

	*U*^11^	*U*^22^	*U*^33^	*U*^12^	*U*^13^	*U*^23^
C1	0.040 (2)	0.040 (2)	0.0315 (18)	0.0004 (17)	0.0049 (17)	0.0066 (16)
O1	0.0461 (15)	0.0741 (16)	0.0302 (13)	0.0064 (12)	0.0012 (12)	−0.0026 (12)
C2	0.037 (2)	0.048 (2)	0.0318 (18)	0.0017 (17)	−0.0009 (17)	0.0046 (16)
C3	0.044 (2)	0.042 (2)	0.0299 (18)	−0.0017 (17)	0.0015 (17)	0.0036 (15)
C4	0.0311 (18)	0.0391 (18)	0.0304 (17)	0.0046 (18)	−0.0007 (16)	0.0061 (17)
C5	0.0268 (19)	0.049 (2)	0.0369 (19)	−0.0029 (16)	−0.0027 (16)	0.0068 (16)
C6	0.036 (2)	0.044 (2)	0.0337 (18)	−0.0008 (17)	0.0029 (17)	0.0035 (16)
Br6	0.0554 (2)	0.0894 (3)	0.0494 (2)	0.0029 (2)	0.00389 (16)	−0.0202 (2)
C7	0.031 (2)	0.037 (2)	0.050 (2)	0.0009 (17)	0.0068 (18)	0.0070 (17)
O7	0.0331 (13)	0.0724 (17)	0.0630 (14)	0.0089 (14)	0.0056 (12)	0.0020 (14)
C71	0.035 (2)	0.075 (3)	0.097 (3)	0.008 (2)	0.004 (2)	−0.001 (2)
C8	0.030 (2)	0.062 (2)	0.050 (2)	−0.0027 (18)	−0.0070 (18)	0.0002 (19)
C9	0.044 (2)	0.060 (2)	0.0341 (19)	0.0031 (19)	−0.0054 (19)	−0.0016 (18)
C11	0.036 (2)	0.0310 (19)	0.0313 (18)	0.0003 (15)	0.0044 (16)	0.0003 (15)
C12	0.044 (2)	0.054 (2)	0.042 (2)	0.0106 (19)	0.0055 (18)	−0.0118 (17)
C13	0.052 (2)	0.057 (2)	0.038 (2)	0.008 (2)	0.0019 (19)	−0.0094 (18)
C14	0.037 (2)	0.035 (2)	0.049 (2)	−0.0003 (16)	0.0010 (19)	0.0040 (17)
C141	0.048 (2)	0.065 (3)	0.064 (3)	0.0112 (19)	−0.003 (2)	0.001 (2)
C15	0.037 (2)	0.052 (2)	0.045 (2)	0.0082 (18)	0.0116 (18)	0.0060 (17)
C16	0.051 (2)	0.047 (2)	0.0275 (18)	0.0027 (19)	0.0032 (18)	0.0027 (16)

### Geometric parameters (Å, °)

**Table:**

C1—O1	1.218 (3)	C71—H71C	0.9600
C1—C2	1.478 (4)	C8—C9	1.388 (4)
C1—C11	1.491 (4)	C8—H8	0.9300
C2—C3	1.326 (4)	C9—H9	0.9300
C2—H2	0.9300	C11—C16	1.383 (4)
C3—C4	1.452 (4)	C11—C12	1.392 (4)
C3—H3	0.9300	C12—C13	1.381 (4)
C4—C5	1.382 (4)	C12—H12	0.9300
C4—C9	1.391 (4)	C13—C14	1.374 (4)
C5—C6	1.374 (4)	C13—H13	0.9300
C5—H5	0.9300	C14—C15	1.386 (4)
C6—C7	1.383 (4)	C14—C141	1.501 (4)
C6—Br6	1.888 (3)	C141—H14A	0.9600
C7—O7	1.358 (3)	C141—H14B	0.9600
C7—C8	1.384 (4)	C141—H14C	0.9600
O7—C71	1.417 (3)	C15—C16	1.366 (4)
C71—H71A	0.9600	C15—H15	0.9300
C71—H71B	0.9600	C16—H16	0.9300
			
O1—C1—C2	120.8 (3)	C7—C8—H8	120.2
O1—C1—C11	119.9 (3)	C9—C8—H8	120.2
C2—C1—C11	119.3 (3)	C8—C9—C4	121.9 (3)
C3—C2—C1	120.8 (3)	C8—C9—H9	119.0
C3—C2—H2	119.6	C4—C9—H9	119.0
C1—C2—H2	119.6	C16—C11—C12	117.3 (3)
C2—C3—C4	129.2 (3)	C16—C11—C1	119.0 (3)
C2—C3—H3	115.4	C12—C11—C1	123.7 (3)
C4—C3—H3	115.4	C13—C12—C11	120.4 (3)
C5—C4—C9	117.4 (3)	C13—C12—H12	119.8
C5—C4—C3	124.0 (3)	C11—C12—H12	119.8
C9—C4—C3	118.6 (3)	C14—C13—C12	121.5 (3)
C6—C5—C4	121.2 (3)	C14—C13—H13	119.2
C6—C5—H5	119.4	C12—C13—H13	119.2
C4—C5—H5	119.4	C13—C14—C15	118.3 (3)
C5—C6—C7	121.2 (3)	C13—C14—C141	120.6 (3)
C5—C6—Br6	120.0 (2)	C15—C14—C141	121.1 (3)
C7—C6—Br6	118.7 (3)	C14—C141—H14A	109.5
O7—C7—C6	117.1 (3)	C14—C141—H14B	109.5
O7—C7—C8	124.1 (3)	H14A—C141—H14B	109.5
C6—C7—C8	118.8 (3)	C14—C141—H14C	109.5
C7—O7—C71	118.0 (3)	H14A—C141—H14C	109.5
O7—C71—H71A	109.5	H14B—C141—H14C	109.5
O7—C71—H71B	109.5	C16—C15—C14	120.1 (3)
H71A—C71—H71B	109.5	C16—C15—H15	119.9
O7—C71—H71C	109.5	C14—C15—H15	119.9
H71A—C71—H71C	109.5	C15—C16—C11	122.4 (3)
H71B—C71—H71C	109.5	C15—C16—H16	118.8
C7—C8—C9	119.5 (3)	C11—C16—H16	118.8

### Hydrogen-bond geometry (Å, °)

**Table:**

*D*—H···*A*	*D*—H	H···*A*	*D*···*A*	*D*—H···*A*
C2—H2···O1^i^	0.93	2.61	3.536 (3)	178
C5—H5···O1^i^	0.93	2.69	3.603 (4)	167
C12—H12···O1^i^	0.93	2.50	3.424 (4)	171
C141—H14B···Br6^ii^	0.96	3.14	4.100 (3)	176

Symmetry codes: (i) *x*, −*y*+1/2, *z*+1/2; (ii) *x*+1, −*y*+1/2, *z*−1/2.
